# N-Acetyltransferase Polymorphism and Risk of Colorectal Adenoma and Cancer: A Pooled Analysis of Variations from 59 Studies

**DOI:** 10.1371/journal.pone.0042797

**Published:** 2012-08-14

**Authors:** Jinxin Liu, Dapeng Ding, Xiaoxue Wang, Yizhi Chen, Rong Li, Ying Zhang, Rongcheng Luo

**Affiliations:** 1 Department of Oncology, Nanfang Hospital, Southern Medical University, GuangZhou, China; 2 Department of Oncology, Longgang District Central Hospital of ShenZhen, ShenZhen, China; 3 Institute of Genetic Engineering, Southern Medical University, Guangzhou, China; 4 Department of Proctology, The Sixth Affiliated Hospital of Sun Yat-sen University, Guangzhou, China; 5 Department of Health Records, Longgang District Central Hospital of ShenZhen, ShenZhen, China; 6 College of Pharmacy, Jinan University, Guangzhou, China; University of Aberdeen, United Kingdom

## Abstract

**Background:**

There have been an increasing number of studies with evidence suggesting that the N-acetyltransferase 1 (NAT1) and N-acetyltransferase 2 (NAT2) genotypes may be implicated in the development of colorectal cancer (CRC) and colorectal adenoma (CRA). So far the published data on this association has remained controversial, however. We performed a meta-analysis of case-cohort and case-control studies using a subset of the published data, with an aim to derive a better understanding of the underlying relationship.

**Methods/Principal Findings:**

A literature search was performed using Medline database for relevant studies published through October 31, 2011. A total of 39 publications were selected for this meta-analysis, including 11,724 cases and 16,215 controls for CRC, and 3,701 cases and 5,149 controls for CRA. In our pooled analysis of all these studies, the results of our meta-analysis suggested that the NAT1 genotype was not significantly associated with an elevated CRC risk (OR 0.99, 95% CI 0.91–1.07). We also found that individuals with the rapid NAT2 genotype did have an elevated risk of CRC (OR 1.07, 95% CI 1.01–1.13). There was no evidence for an association between the NAT1 and 2 rapid genotype and an elevated CRA risk (NAT1: OR 1.14, 95% CI 0.99–1.29; NAT2: OR 0.94, 95% CI 0.86–1.03).

**Conclusion:**

This meta-analysis suggests that individuals with NAT2 genotype had an elevated risk of CRC. There was no evidence for the association between NAT1 and 2 rapid genotype and CRA risk.

## Introduction

Meat consumption has been linked to an increased risk of colorectal cancer (CRC) in many epidemiological studies [1], [2]. Potent mutagens such as heterocyclic amines (HCAs) and polycyclic aromatic hydrocarbons (PAHs) are formed during the high-temperature cooking of meat. The chemical structure of the HCA can be detoxified by the phase II enzymes N-acetyltransferase 1 and 2 (NAT1 and NAT2). The alteration of NAT1 and NAT2 acetylator status may decrease enzymatic activity and thus lead to a decreased efficiency in detoxification in the body, further resulting in an elevated risk of cancer.

So far, 36 NAT2 genetic variants have been identified in human, of which NAT2*4 is the most common allele associated with rapid acetylation [3]. Meanwhile, NAT2*11A, NAT2*12A-C, NAT2*13A and NAT2*18 are also classified as rapid alleles, while the rest of the alleles are considered as slow alleles. Four relatively common polymorphic alleles exist for NAT1: designated as NAT1*3, NAT1*4, NAT1*10, and NAT1*11, with NAT1*4 being the most common allele and NAT1*10 the putative rapid allele. Subjects with more than one rapid allele were classified under NAT1 rapid acetylation, while others were under NAT1 slow acetylation.

Previous studies have investigated the relationship between the NAT1 and NAT2 genotype and predisposition to CRC and CRA [4]–[43]. The results were, however, inconsistent and even contradictory. Each individual study may have been underpowered to detect the effect of NAT1 and NAT2 genotype on the susceptibility of CRC and CRA. We therefore performed this meta-analysis of all eligible studies to derive a more scientifically convincing association of the NAT1 and NAT2 genotype with CRC and CRA.

## Methods

### Study eligibility, criteria, and literature searches

**Figure 1:**
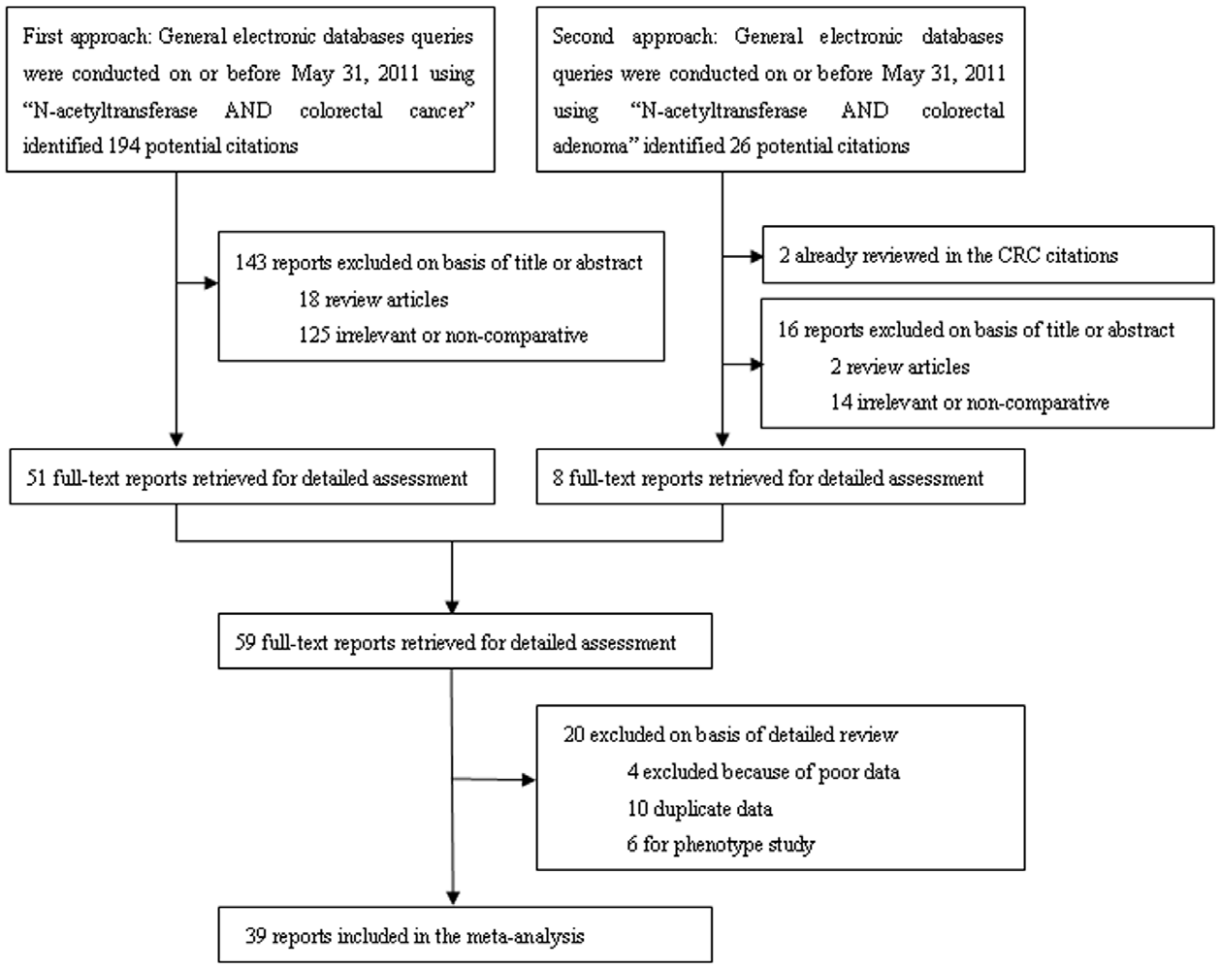
Studies identified with criteria for inclusion and exclusion.

Computerised searches in MEDLINE were performed using the following search terms “NAT1”, “NAT2”, “genotype”, and “colorectal cancer” or “colorectal adenoma” (the last search update was October 31, 2011). As studies with the same population by different investigators or overlapping data by the same authors were found, the most recent or complete articles with the largest numbers of subjects were included. Abstracts written in non-English language were not considered. Our initial search and the process of study selection is summarised in Figure 1.

### Inclusion criteria

All human-associated studies were included if they met the following criteria: (1) evaluate the association between NAT1 and NAT2 genotype and the risk of CRC and CRA; (2) CRC and CRA cases must have been diagnosed by histological examination; (3) studies with full-text article.

### Quality assessment

**Figure 2:**
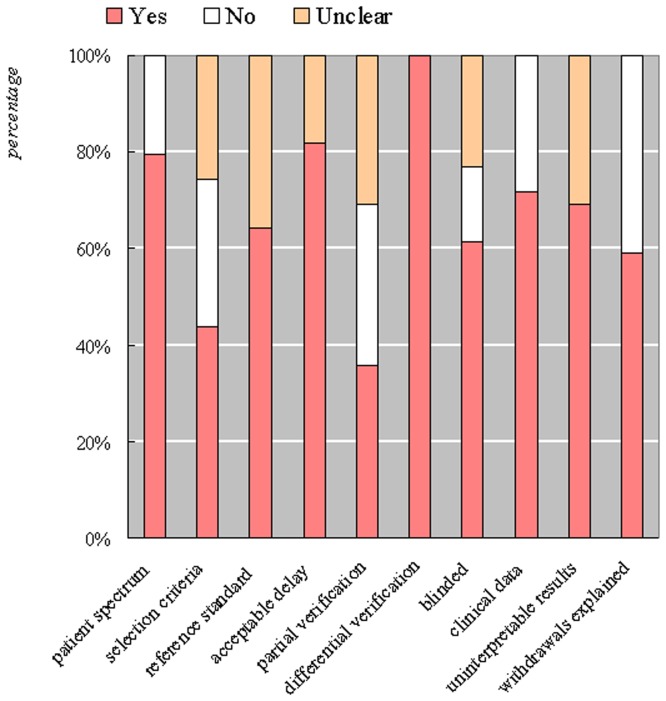
Results of quality assessment for appropriate spectrum studies.

Study quality was assessed using the Quality Assessment of Diagnostic Accuracy Studies (QUADAS) checklist (shown in Figure 2). Of the fourteen items in the checklist, ten items were relevant to this review and were used [44].

### Data extraction

Data were extracted from each study by two reviewers independently according to the prespecified selection criterias (shown in Table 1). Supplementary information was obtained, when required, by contacting the lead author of the study. Contact was made by mail. In total, the lead authors of four studies [45]–[48] were contacted, but none replied. The following characteristics were collected for each study: first author's name, ethnicity, source of case and control groups (hospital patient, healthy individual, population control, and random individual).

**Table 1:** Characteristics of studies included in the meta-analysis.

Study	Place of study	Gene	Ethnicity	Case	Case comment	Control	Design
Bell et al. 1995	UK	NAT1, 2	Caucasian	202	CRC cases with adenocarcinoma of colon or rectum from the North Staffordshire Hospital between 1990–1994	112	HP
Probst-Hensch et al. 1996	USA	NAT1, 2	Mixed	441	CRA cases from two Southern California Kaiser Permanente Medical Centers between 1991 and 1993	484	PB
Welfare et al. 1997	UK	NAT2	Caucasian	174	CRC cases in the Newcastle and North Tyneside health districts over a 9 month period	174	PB
Chen et al. 1998	USA	NAT1, 2	Caucasian(98%)	212	Male confirmed of CRC cases from the Physicians' Health Study	221	HI
Hubbard et al. 1998	UK	NAT2	Caucasian	245	CRC sample collected from three local hospitals between 1988 and 1993	217	HI
Gil and Lehner et al. 1998	Portugal	NAT2	Caucasian	114	CRC cases from the Lisbon area or South/Central Portugal during the period 1994 to 1996	201	PB
Lee et al. 1998	Singapore	NAT2	Asian	216	CRC cases recruited from the National University Hospital and Singapore General Hospital	187	HI
Kampman et al. 1999	USA	NAT2	Caucasian	1,624	CRC cases with the primary colon carcinoma diagnosed between 1991 to 1994	1,963	PB
Potter et al. 1999	USA	NAT2	Caucasian	527	CRA cases from an NCI-funded program project between 1991 and 1994	633	PB
Katoh et al. 2000	Japan	NAT1, 2	Asian	103	CRC cases with colorectal adenocarcinoma from Kitakyushu City during 1991 to 1995	122	RI
Agúndez et al. 2000	Spain	NAT2	Caucasian	120	CRC cases with carcinoma of colon or rectum from 1997 to 1999	258	HI
Ishibe et al. 2002	USA	NAT1, 2	Mixed	146	CRA cases from a clinic–based case–control study	228	HP
Tiemersma et al. 2002	Netherlands	NAT1, 2	Caucasian	102	CRC cases from Netherlands Cancer Registry (NCR) and three regional cancer registries between 1987 to 1998	536	RI
Barrett et al. 2003	Sweden	NAT2	Caucasian	490	CRC cases recruited from three centres: Dundee, Leeds and York in the period 1997–2000	592	PB
Van der Hel et al. 2003	Netherlands	NAT1, 2	Caucasian	258	Female with diagnosis of CRC cases recruited from 1987 to 1996	857	PB
Kiss et al. 2004	Hungary	NAT2	Caucasian	500	CRC cases from Centre Hospital of Ministry of Internal Affairs and from the area of Baranya and Vas Country	500	PB
Tiemersma et al. 2004	Netherlands	NAT1, 2	Caucasian	431	CRA cases recruited among patients at the eight hospitals in the Netherlands between 1997 and 2000	433	HP
He et al. 2005	China	NAT2	Asian	83	CRC cases recruited from the Department of Surgery at Hebei No. 4 Hospital	237	HI
Chan et al. 2005	USA	NAT2	Caucasian	183	183 female cases with CRC from the Nurses' Health Study from 1976 in 11 US states	443	HP
Landi et al. 2005	Spain	NAT1, 2	Caucasian	360	CRC cases from the University Hospital in Barcelona from 1996 to 1998	308	HP
Chen et al. 2005	China	NAT1, 2	Asian	139	CRC cases from the population census in ZheJiang province from 1989 to 1990	343	HI
Moslehi et al. 2006	USA	NAT2	Mixed	685	CRA cases from screening–arm participants of the PLCO Trial between 1993 and 1999	693	PB
Lilla et al. 2006	Germany	NAT1, 2	Caucasian	505	CRC cases from 22 hospitals in the Rhine-Neckar-Odenwald region between 2003 and 2004	604	PB
Borlak et al. 2006	Germany	NAT2	Caucasian	92	CRC cases (colon) provided by the the Imperial Cancer Research Fund Laboratory of the Ninewell's Hospital	243	HI
Pistorius et al. 2006	Germany	NAT2	Caucasian	209	Patients with diagnosis of sporadic or familial CRC cases who met at least one criterion of the Bethesda guidelines	100	HI
Huang et al. 2007	China	NAT2	Asian	244	CRC cases recruited from the Chung Shan Medical University Hospital from 2000 to 2005	299	PB
Mahid et al. 2007	USA	NAT1, 2	Caucasian	122	Sporadic CRC cases from the University of Louisville colorectal surgery unit	222	RI
Yoshida et al. 2007	Japan	NAT2	Asian	66	CRC cases from the Kobe Medical Center and Rosai Hospital between 2003 and 2005	121	RI
Butler et al. 2008	USA	NAT1, 2	Mixed	507	217 African cases and 290 Caucasian cases with adenocarcinoma of colon cancer between 1996 and 2000	849	PB
Shin et al. 2008	USA	NAT1, 2	Mixed	557	CRA cases from Tennessee Colorectal Polyp Study (TCPS)	1,493	HP
S???rensen et al. 2008	Denmark	NAT2	Caucasian	377	CRC cases from a Danish prospective study from 1993 to 2003	768	PB
Cotterchio et al. 2008	Canada	NAT2	Caucasian	832	CRC cases from the OFCCR between 1997 and 2000	1,247	PB
Yeh et al. 2009	China	NAT1, 2	Asian	727	CRC cases recruited from the Chang Gung Memorial Hospital between 1995 and 1999	736	HI
Nöthlings et al. 2009	USA	NAT1, 2	Mixed	1,009	CRC cases from the Multiethnic Cohort Study between 1993 and 1996	1,522	PB
Zupa et al. 2009	Italy	NAT2	Caucasian	92	CRC cases of colon cancer from the Centro di Riferimento Oncologico of Basilicata	121	HI
Wang et al. 2010	USA	NAT2	Mixed	914496	CRA cases from Hawaii during 1996 to 2007 and CRC cases from Hawaii during 1994 to 1999	1,185607	HP
Da Silva et al. 2010	Brazil	NAT2	Mixed	147	CRC cases from Department of Gastroenterology, University Hospital between 2008 and 2009	212	HI
Cleary et al. 2010	Canada	NAT1	Caucasian	1,174	CRC cases from the OFCCR between 1997 and 2000	1,293	PB

HB, hospital–based patient; PB, population-based control; HI, healthy individual; RI, random individual.

### Statistical methods

Statistical analyses were done with Stata, version 11.0. Heterogeneity among studies was checked by the random-effects model (the DerSimonian and Laird method) if there was significant heterogeneity. A *P* value of more than the nominal level of 0.05 for the Q statistic indicated a lack of heterogeneity across studies, allowing the use of the fixed-effects model (the Mantel-Haenszel method). Subgroup analyses were performed by type of study, genotype (NAT1 and NAT2), ethnicity, and source of control. Funnel plot asymmetry was assessed by the method of Egger's linear regression test. The significance of the intercept was determined by the *t* test suggested by Egger (*P*<0.05 was considered representative of statistically significant publication bias).

## Results

### Eligible studies and meta-analysis database

As summarised in Table 1, 39 publications were selected for this meta-analysis, including 48 studies of the colorectal cancer (11,724 CRC cases and 16,215 controls) and 11 studies of the colorectal adenoma (3,701 CRA cases and 5,149 controls). CRC and CRA cases were recruited from 15 countries from 1995 to 2010. And subjects of controls were matched for age or gender.

### Quantitative synthesis

Table 2 lists the main results of the meta-analysis for colorectal cancer. In total, the carrier frequency of the NAT1 rapid genotype was 47.1% in cases with CRC and 45.9% in controls. And for NAT2 genotype, the carrier frequency of rapid genotype was 53.1% in cases with CRC and 51.1% in controls. Overall, no significant associations were found between NAT1 rapid genotype and CRC risk when all studies pooled into the meta-analysis (OR 0.99, 95% CI 0.91–1.07). And there was no statistically heterogeneity among these studies in overall comparisons (*P* = 0.31, *I^2^* = 13.5%). In the subgroup analyses by ethnicity, no significant associations were found in all comparisons (OR 1.01, 95% CI 0.92–1.12 for Caucasian; OR 1.10, 95% CI 0.91–1.34 for Asian; OR 0.85, 95% CI 0.85–1.07 for African).

**Table 2:** Stratified analysis of the NAT1, NAT2 genotype on colorectal cancer and adenoma risk.

Type of study	No	Sample (cas/con)	Test of association				Test of heterogeneity		
			OR	95% CI	P	Result	Q	P	I^2^ (%)
**Colorectal cancer**									
**NAT1 acetylator**	14	5,177/7,475	0.99	0.91–1.07	0.74	−	15.03	0.31	13.50
Ethnicity									
African	1	208/299	0.95	0.85–1.07	0.40	−	_	_	**_**
Asian	3	963/1,198	1.10	0.91–1.34	0.32	−	0.26	0.88	0.00
Caucasian	9	3,162/4,633	1.01	0.92–1.12	0.80	−	10.15	0.26	21.20
Mixed	1	844/1,345	0.94	0.87–1.01	0.09	−	_	_	**_**
Source of control									
Healthy individual	3	1,072/1,297	1.08	0.90–1.29	0.42	−	0.64	0.73	0.00
Hospital patient	2	561/433	1.44	1.06–1.94	0.02	+	2.23	0.14	55.10
Population control	6	3,216/4,864	0.94	0.86–1.03	0.19	−	3.28	0.66	0.00
Random individual	3	328/881	0.91	0.69–1.19	0.48	−	0.69	0.71	0.00
**NAT2 acetylator**	34	10,509/14,964	1.07	1.01–1.13	0.01	+	46.70	0.06	29.30
Ethnicity									
African	1	215/307	1.01	0.88–1.16	0.91	−	_	_	**_**
Asian	7	1,566/2,039	1.12	0.94–1.33	0.20	−	5.64	0.47	0.00
Caucasian	23	7,096/10,307	1.09	1.02–1.16	0.01	+	36.04	0.03	39.00
Mixed	3	1,632/2,311	0.96	0.84–1.10	0.56	−	2.05	0.36	2.60
Source of control									
Healthy individual	12	2,270/2,969	0.93	0.82–1.05	0.25	−	11.74	0.38	6.30
Hospital patient	4	1,283/1,469	1.04	0.88–1.23	0.66	−	0.34	0.95	0.00
Population control	14	6,608/9,525	1.14	1.03–1.26	0.01	+	26.28	0.02	50.50
Random individual	4	393/1,001	1.06	0.79–1.41	0.72	−	1.46	0.69	0.00
**Colorectal adenoma**									
Genotype									
NAT1	4	1,553/2,587	1.14	0.99–1.29	0.06	−	1.29	0.73	0.00
NAT2	7	3,683/5,109	0.94	0.86–1.03	0.18	−	9.83	0.13	39.00
Ethnicity									
Caucasian	3	1,381/1,496	1.07	0.92–1.25	0.36	−	2.21	0.33	9.40
Mixed	8	3,855/6,200	0.98	0.90–1.06	0.55	−	13.12	0.07	46.60
Source of control									
Hospital patient	7	3,142/5,402	1.02	0.93–1.11	0.72	−	10.46	0.11	42.60
Population control	4	2,094/2,294	0.96	0.86–1.09	0.54	−	5.57	0.14	46.10

Based on meta-analysis from 34 studies, a significant increased risk of colorectal cancer was observed for those individuals with rapid acetylator polymorphisms in NAT2 (OR 1.07, 95% CI 1.01–1.13). When stratified by ethnicity, elevated risk of CRC was also observed among Caucasian populations (OR 1.09, 95% CI 1.02–1.16). There was no evidence for the association between the rapid genotype and CRC risk in Asian and African. And in a stratified analysis by source of control, individuals carrying the NAT2 rapid genotype were not significantly associated with increased CRC risk. There was no statistically heterogeneity among these studies in overall comparisons (*P* = 0.06, *I^2^* = 29.3%).

The associations of CRA risks with NAT1 and NAT2 genotype are also shown in Table 2. There was no evidence for the association between NAT1 and 2 rapid genotype and CRA risk (NAT1: OR 1.14, 95% CI 0.99–1.29; NAT2: OR 0.94, 95% CI 0.86–1.03). Similarly, no associations were found for the stratified analysis by ethnicity.

### Publication bias

**Figure 3:**
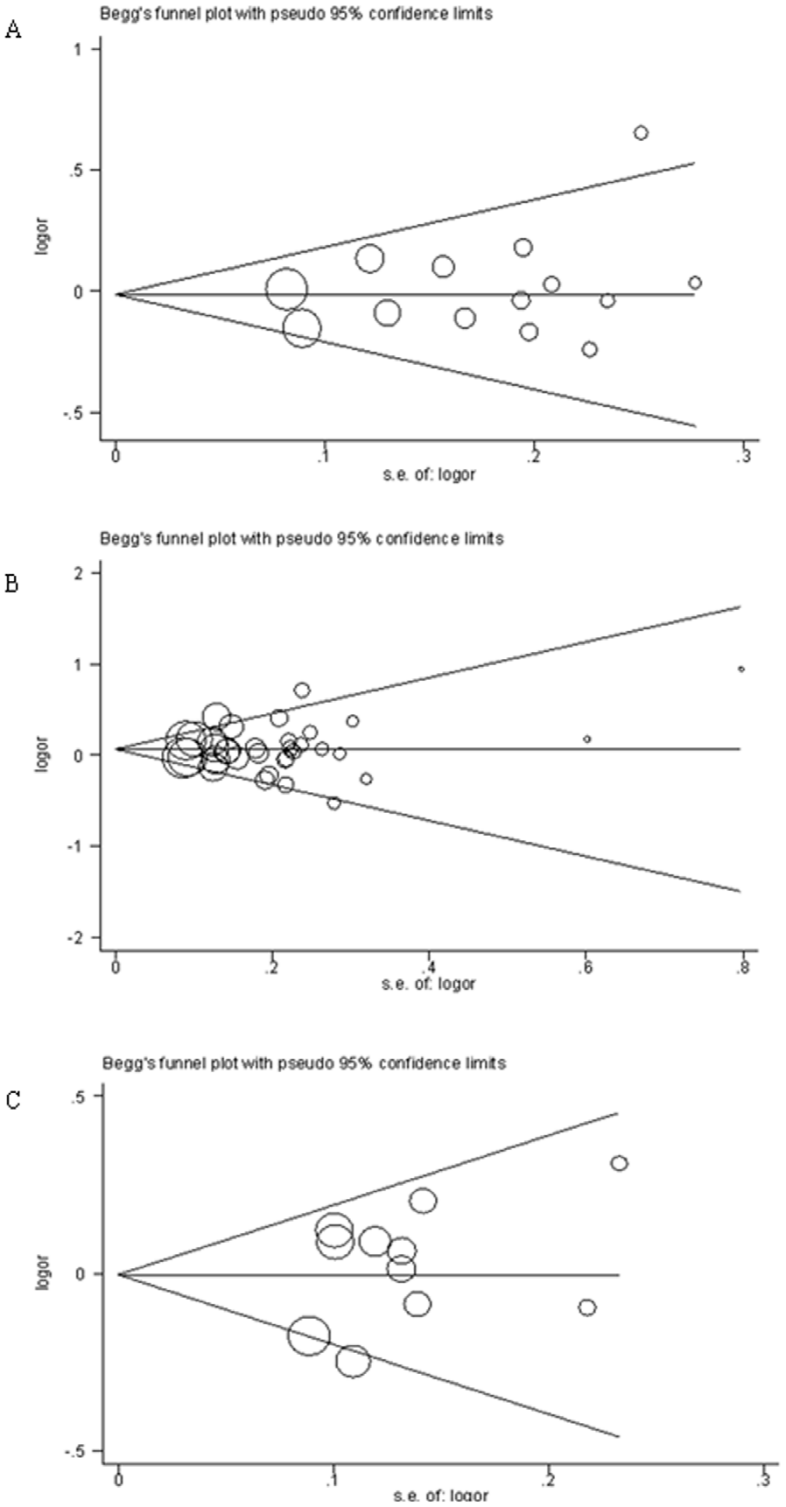
Begg's funnel plot for publication bias test: Rapid versus slow. Each point represents a separate study for the indicated association. Log (OR), natural logarithm of OR. Horizontal line: mean effect size. A: Funnel plot analysis for odds ratios for NAT1 genotype in overall CRC studies; B: Funnel plot analysis for odds ratios for NAT 2 genotype in overall CRC studies; C: Funnel plot analysis for odds ratios for NAT1 and NAT2 genotype in overall CRA studies.

We performed Begg's funnel plot and Egger's test to assess the publication bias of literatures. As shown in Figure 3, the shape of the funnel plots did not reveal any evidence of obvious asymmetry. And the results of Egger's test did not suggest any evidence of publication bias (*P* = 0.353 for NAT1 genotype with CRC, *P* = 0.931 for NAT2 genotype with CRC, *P* = 0.183 for NAT1 genotype with CRA, *P* = 0.802 for NAT2 genotype with CRA). Although the sample size for cases and controls in 39 publications ranged from 66 to 1,963, the corresponding pooled ORs were not qualitatively altered with or without the study of small sample.

## Discussion

Allelic polymorphism of the NAT2 enzyme has been studied for a long time, first detected phenotypically, based on enzyme activity distribution in healthy subjects, and later these activity differences were bound to an allelic polymorphism [49]. NAT1 was originally believed to be monomorphic, because of the unimodal distribution of its activity in the studied populations. Numerous studies have investigated the relationship between NAT1 and NAT2 genotypes and CRC or CRA susceptibility. The results vary widely and are often discordant likely because of ethnic and geographic differences of the enrolled subjects. In order to resolve this conflict, this meta-analysis was performed to derive a more precise estimation of the association.

Variations in the frequency of NAT1 and NAT2 genotype among different ethnic groups have been reported. Our results showed that frequency of the NAT1 rapid acetylation phenotype was 65.4% in Asian populations, which was significantly higher than that in European populations (35.8%). And for NAT2 phenotype, the rapid acetylation phenotype frequency was 79.8% in Asians and 42.4% in Europeans. In the stratified analysis by ethnicity, elevated risk was found for Caucasian. But there was no evidence for the association between NAT1 and NAT2 genotype and CRC risk among Asian populations.

There is epidemiologic evidence for differential effects of acetylation with CRA risk by ethnicity. For example, Probst-Hensch et al. reported an inverse association between NAT2 rapid genotypes and colorectal adenomas among African Americans, but an increased risk among whites [50]. It has been suggested that the individual single nucleotide polymorphisms may occur uniquely within specific ethnic populations resulting in allele frequencies and could account for racial differences in disease susceptibility [51]. For a long time, genetic susceptibility to cancer has been attributed to xenobiotic exposure. However, this view has changed with the advances in molecular biology. It is now known that exposure to xenobiotics and the development of cancer vary among individuals because of variations that occur at the molecular level which, in turn, are under genetic control [52]. In recent studies, lifestyle habits including alcohol and tobacco use and dietary habits (meat intake) have been associated with gene mutations in an attempt to obtain more consistent results regarding cancer risk factors and prognosis [53]. Although currently available data are controversial due to ethnic differences and differences in lifestyle, this has been the best approach to better understand carcinogenesis at the molecular level.

Several studies have also investigated the association between NAT1 and NAT2 genotypes and CRC risk, as reviewed in 2000 [4]. Contrary to the negative result of most articles, some findings suggest that NAT2 gene variants associated with more rapid acetylation activity may be related to increased risk of colorectal cancer [20]. This could be explained by the reaction of meat consumption and cigarette smoking on individuals with high genetic susceptibility. It was reported that NAT2 rapid acetylator genotypes may contribute to CRC risk of individuals with high consumption of red meats, not to that of active smokers [28]. However, a meta-analysis with 20 publications cited that NAT2 rapid acetylation status has no specific effect on the risk of developing colon cancer [54]. The discrepancy could be attributed to the potential problem of misclassification for meat intake and smoking history. There were some other studies on the combined effect of NAT1 or NAT2 and meat intake. However, they did not categorise on how meat was consumed [38], and present information for meat consumption in a uniform standard [28], [35], [39]. Possible misclassification in exposure measurement and heterogeneity in definition of meat intake or tobacco use may partly explain the high inconsistency of the findings.

The present study has several limitations that need consideration. First, we only considered two metabolic enzymes (NAT1 and NAT2). Because additional enzymes are involved in the bioactivation and detoxification of heterocyclic amine, they may also play a role in modifying CRC or CRA risk, this may increase the misclassification of measured variables. Second, only three studies evaluated associations between NAT1 or NAT2 genotype and CRC risk in histologic subgroup, such as associations among cases in the Duke stage [5], [14], [28]. Thirdly, our meta-analysis was based on unadjusted OR estimates because not all published studies were presented with adjusted ORs [15], [42] or when they did, the ORs were not adjusted by the same potential confounders [16], [22], [27]. The magnitude of the observed association for NAT2 with CRC risk is modest, possibly owing to the unadjusted estimate by age and sex in the pooled adjusted analysis. Given these results, our conclusions should be interpreted cautiously.

In conclusion, our meta-analysis suggests that individuals with the rapid NAT2 genotype had elevated risk of CRC. There was no evidence for the association between NAT1 and 2 rapid genotype and CRA risk. Our study significantly increased the statistical power of the analysis based on the studies for CRC and CRA risk. Further studies on estimating the effect of gene-gene and gene-environment interactions may eventually lead to a better and more comprehensive understanding of the association between the NAT1 and NAT2 genotype and CRC and CRA risk.
